# Prevalence of color blindness among school children in three primary schools of Gish –Abay town district, Amhara regional state, north-west Ethiopia

**DOI:** 10.1186/s12886-018-0970-4

**Published:** 2018-11-26

**Authors:** Mengistu Zelalem Wale, Yekoye Abebe, Yilikal Adamu, Abebe Zelalem

**Affiliations:** 1 Department of Medical Physiology, School of Medicine, College of Health Sciences, Addis Ababa University, Addis Ababa, Ethiopia; 2 Department of Ophthalmology, School of Medicine, College of Health Sciences, Addis Ababa University, Addis Ababa, Ethiopia; 3 Department of Nursing, College of Medicine and Health Sciences, Arba Minch University, Arba Minch, Ethiopia

**Keywords:** Prevalence, Color blindness, Visual impairment, School children, Ethiopia

## Abstract

**Background:**

Although there are limited studies, recent data are lacking to accurately determine the magnitude of color blindness in Ethiopia and there is no evidence of such a study in Gish Abay town district. The purpose of thie study was to assess the prevalence of color blindness among school children in Gish Abaya town district, Ethiopia.

**Methods:**

The study used a community-based analytical cross-sectional study design with multistage cluster random sampling technique from September to October 2016. Three primary schools were selected randomly in the district of Gish Abay town district. Ishihara color plates (24 –edition) was used for color vision test and Snellen’ tumbling ‘E’ chart was used for visual acuity test. The data was analyzed using Statistical Package for Social Sciences (SPSS) version 20 statistical software and binary logistic regression was used to identify factors associated with color blindness.

**Results:**

Among a total of 854 subjects, 850 participants with age range of 8–18 years were screened for color vision test giving a response rate of 99.53%. Among the participants, 452 (53.2%) were males and 398 (46.8%) were females. There were 36 (4.24%) cases of impaired color vision. Among these, 27 (3.18%) were males and 9 (1.06%) were females. Out of 36 cases of color blindness, 15 (1.77%) were deutan, 7 (0.82%) were protan and 14(1.65%) were unclassified (both deutan and protan forms). The variables; sex adjusted odds ratio (AOR [95% Confidence Interval] =3.19 [1.45; 6.98], *p*-value = 0.004); and visual impairment (AOR [95% CI] =4.15 [1.77; 9.75], *p*-value = 0.001) were significantly associated with color impairment.

**Conclusion:**

The prevalence of childhood color blindness in Gish Abay town district was relatively similar with other studies in Ethiopia. Sex and visual impairment are factors found to be related with the children’s color blindness. Periodical eye examination at the time of school admission is recommended to adjust the children’s occupation early in life.

## Background

Color blindness is the inability to clearly differentiate color differences under normal lighting conditions. Trichromatic theory of color vision in humans is based on unequal stimulation of the three classes of cones to light of different wave lengths [1]. There are three physiological substrates for normal color vision in humans. These are the short- (S-), medium- (M-), and long- (L) wavelength sensitive cones [2]. Mutations and rearrangements in the genes encoding the the three classes of cone pigments results color blindness [1].

The two broad categories of color blindness are red/protan and green/deutan defects. Protan and deutan defects are characterized by an absence or anomaly of L-cone, and M- cone function respectively. Deuteranopia arises due to the absence of photo pigment of the green cone; whereas, Protanopia arises due to the absence of red cone [1, 3]. John Dalton was the first scientist to give a clear description of his own affliction of color blindness in 1798 [4].

Since mostly color blindness is a genetic disorder, the incidence varies from race to race and across different geographic araeas [4, 5]. In addition to hereditary conditions, color blindness occurs due to acquired conditions such as ocular diseases or injury or disease of retina by trauma, chronic diseases, drugs, toxins, alcoholism, and aging. Acquired defects are the less common forms and are not associated with the alteration of opsin genes [6].

Eventhogh color blindness is not physically debilitating, it can have a major impact on one’s day-to-day life. Color blind persons may not be able to differentiate between red and green traffic signals. They may also face difficulties at work as seen in technician working in color industries [7].

In different areas across the world, the prevalence of color blindness varies [5, 8]. In a study conducted in north-western Ethiopia among school children, the prevalence of color blindness was found to be 4.2% in males and 0.2% among females [9]. In another study conducted among male school children in Ethiopia, the prevalence of color blindness was 4.2% [3], in Pokhara, Western Nepal it was 3.8% [4], in India, 3.7% [8], in Philippines, 5.17% [10], and in Italy the prevalence was found to be 5.91% [11].

Our study determined the prevalence of color blindness, among school children in three primary schools of Gish Abay town district, north-west**,** Ethiopia. Determining the magnitude of color blindness is important to adjust their occupation early in life. This may also help professionals to give emphasis on the complications associated with color blindness. This study also identified demographic factors associated with color blindness. This finding may serve as base line data for future related researches in the area.

## Methods

This study was conducted in the district of Gish-Abay town district from September to October 2016. Gish Abay (the place where Abay River originates) is one of the most remote areas in West Gojjam Zone, Amhara Regional State, north-west Ethiopia. Community-based analytical cross-sectional study design was conducted using multistage cluster random sampling technique. The procedure of sample selection was as follows; three primary schools among 15 in the district of Gish-Abay town were selected randomly. Next, the total sample size was distributed proportionally to the selected schools.

Then the total samples of each school were again distributed to each grade (children’s class level) proportionally. Finally, students in each grade were selected using systematic random sampling methods. All school children in Gish-Abay town district were the source population and those who satisfying the study inclusion criteria of the selected schools were the study populations.

All school children in the three primary schools of Gish-Abay town district with age range of 8–18 years were included in this study. Students who can read numbers in the chart, those who were from grades three to eight and who are not bilaterally blind were included for color vision test. Students below grade three, students below 8 and above 18 years of age were excluded for both color vision and visual acuity test. Children with bilateral blindness even with light perception were excluded from color vision test.

The minimum required sample size for this study was obtained using single proportion formula by taking the prevalence of 4.2% from previous study in Ethiopia [3], 2.5% margin of error (possible maximum error) with a design effect (g) of 3 with 95% confidence.

n =  g × Z^2^ × P × (1 ‐ P)

d^2^

n = the final sample size ≅854

P = proportion from previous studies = 0.042

Z = 1.96 at 95% Confidence Interval (CI)

d = Possible maximum error = 0.025

The final sample size was estimated to be 854 (455 males and 399 females) with 15% adjustment for non-response rate. Before administering the test, general instructions were given to the study subjects including the benefits of being examined. Color vision was tested using Ishihara pseudo-chromatic color plate test (24- edition). The test was conducted by the principal investigator and an optometrist in a room with optimum natural daylight hours as recommended by Ishihara guide lines. As described in other studies [3, 5], the distance between the subjects being examined and the chart was 75 centemeters (cm). The test was performed under binocular viewing conditions. As clearly stated in the Ishihara 24-plate edition guide line, the time taken in each plate test was not more than 3 s delay.

To determine the normality or defectiveness of color vision, out of 24 Plates, 1–15 were stated. If 13 or more plates are read normally, the color vision is regarded as normal. If 9 or less plates are read normally, the color vision is regarded as color blind [Ishihara 24-plate edition guideline]. In reference to Plates 14 and 15, only those who read the numerals, 5 and 45 and read them easier than those on 10 and 9 are regarded as abnormal readings. Plate numbers 16 and 17 are used to determine the presence of protanopes and deuteranopes.

All subjects undertaking color vision test under went visual acuity test. The test was conducted using Snelln tumbling ‘E’ chart at 6 m from the observer under monocular viewing in day light hours. The right eye was tested first and then the left one followed. The eye that was not tested was covered with the subject’s hand, ensuring that the subject is not pressing the eye.

The variables age, sex, grades (literacy label) and visual acuity status of the school children were associated with color blindness by using adjusted odds ratio. The data was entered and analyzed using Statistical Package Social Sciences (SPSS) version 20 software and binary logistic regression was used to identify factors associated with color blindness.

### Operational definitions

- *Color blindness*: Those who read 9 or less plates out of 15[Ishihara guide line, 24 plate edition, 3].
- *Visual impairment*: A presenting visual acuity of ≤6/12 in the better eye [12].
- *Normal color vision*: Those who read 13 or more plates out of 15 [12].
- *School children*: Individuals whose age is between 8 and 18 years of old.

## Results

**Fig. 1:**
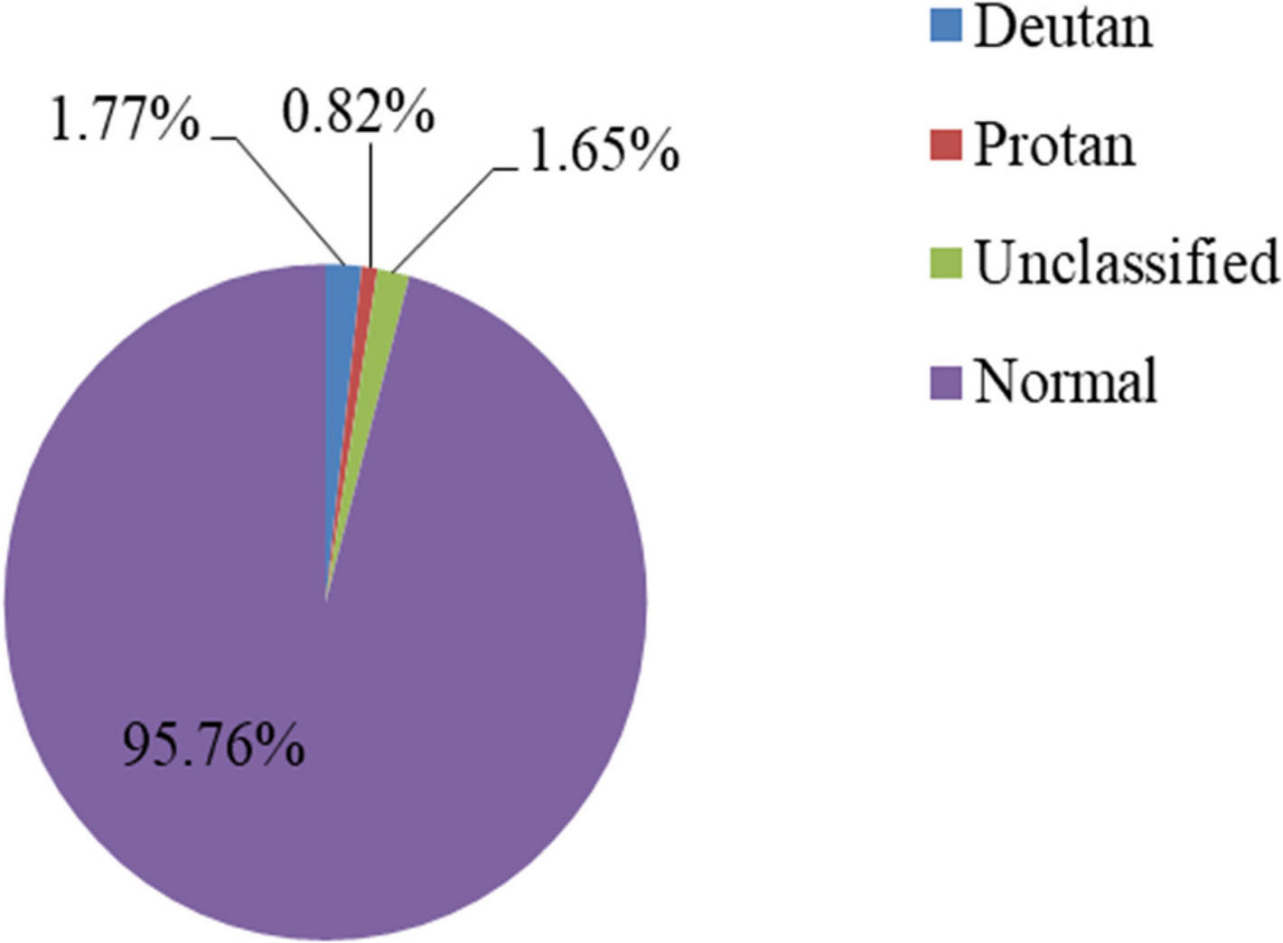
Frequency of color blindness in the three primary schools of Gish- Abay town district, Amhara Regional State, Ethiopia, 2016

From a total of 854 samples, 850 subjects were screened for color vision test giving a response rate of 99.53%. Among the subjects, 452(53.2%) were males and 398(46.8%) were females. The study participants have a mean age of 13.26 years with a standard deviation of ±2.077 years, which ranges from 8 to 18 years. Out of 850 school children who participated for color vision test, 36 (4.24%) were color blind. The distribution of color blindness in the three schools with age, sex and literacy level is shown in (Table 1). Out of 36 color blind students, 15(1.77%) were deutan, 7 (0.82%) were protan and 14 (1.65%) were unclassified (combined form) (Fig. 1).

**Table 1:** The frequency of color blindness among male and female school children in the three primary schools of Gish Abay town district, Amhara Regional State, Ethiopia, 2016

Variables		School name			Total	
		Zeleke Desta % (*n* = 36)	Gumbella % (*n* = 36)	Hamus Wonze % (*n* = 36)		
					% (*n* = 36)	% (*N* = 850)
Literacy Level /Grades/	3rd to 4th	6 (16.7%)	4 (11.1%)	4 (11.1%)	14 (38.9%)	1.65%
	5th to 8th	5 (13.9%)	9 (25%)	8 (22.2%)	22 (61.1%)	2.59%
	Total	11 (30.6%)	13 (36.1%)	12 (33.3%)	36 (100%)	4.24%
Sex	M	7 (19.5%)	10 (27.8%)	10 (27.8%)	27 (75%)	3.18%
	F	4 (11.1%)	3 (8.3%)	2 (5.5%)	9 (25%)	1.06%
Age	8–13	9 (25%)	11 (30.6%)	5 (13.9%)	25 (69.4%)	2.94%
	14–18	2 (5.6%)	2 (5.6%)	7 (19.4%)	11 (30.6%)	1.3%

**Fig. 2:**
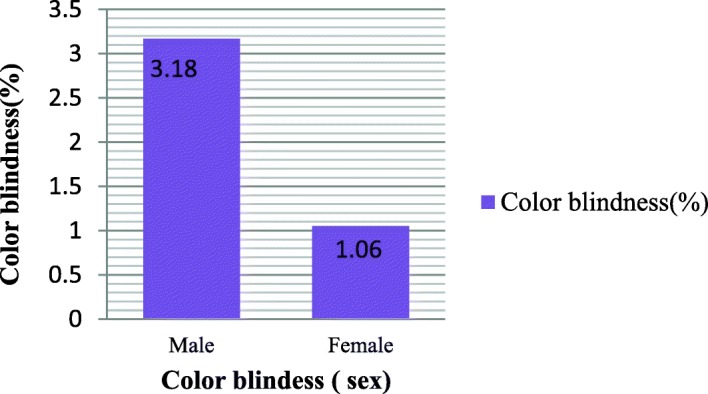
Sex based difference of color blindness in the three primary schools of Gish-Abay town district Amhara Regional State, Ethiopia, 2016

Being an X-linked recessive trait, the prevalence of color blindness with respect to sex is found to be high in males than females **(**Fig. 2). There was a highly significant association between sex and color blindness (*p* =0.001) (Table 2).

**Table 2:** Multiple binary logistic regression analysis of factors associated with color blindness, in the three primary schools of Gish Abay town district, Amhara Regional State, Ethiopia, 2016

Variables		Color vision				OR (95% CI)	*P*-value
		Normal		Color blind			
		*n*	%	*n*	%		
Sex	F	390	45.78%	9	1.05%	1	0.004**
	M	424	50%	27	3.17%	3.19 (1.45–6.98)	
Age	8–18	814	95.78%	36	4.22%	0.805 (0.68–0.94)	0.008**
Grade	3–8	814	95.78%	36	4.22%	0.9 (0.65–1.25)	0.54
VI	No	752	88.5%	28	3.3%	1	0.001**
	Yes	62	7.28%	8	0.92%	4.15 (1.77–9.75)	

Legend: *Significant at 95% level of significance, **significant at 1% level of significance, 1 = reference; *VI* Visual Impairment, *OR* Odds Ratio, *CI*, Confidence Interval

The abnormal readings among 36 color blind subjects, i.e. those who read plate number 14 and 15 easier than 10 and 9 were 21 (58.3%). In reference with plates 16 and 17, from the total cases, 9 (1.05%) were strong deuteranopes (8 males, 1 female) and 4 were strong protanopes (4 males and no females) (Table 3).

**Table 3:** Summary of color vision test from plate 1–17 in the three primary schools of Gish Abay town, Amhara Regional State, Ethiopia, 2016

Charts	1	2	3	4	5	6	7	8	9	10	11	12	13	14	15
Nos	12	8	29	5	3	15	74	6	45	5	7	16	73	***×***	***×***
Read correctly	850	840	811	833	728	822	689	825	803	830	834	823	566	802	801
Misread	–	1	29	5	105	8	131	9	32	6	4	14	269	1	3
RGB no.	12	3	70	2	5	17	21	***×***	***×***	***×***	***×***	***×***	***×***	5	45
RGB Read	–	9	8	10	13	17	28	16	15	14	12	13	15	47	46
C.weaknes	–	–	2	2	4	3	2	–	–	–	–	–	–	–	–
Chart nos	Nos		Read correctly			Misread			Strong deutans				Strong protans		
16	26		832			5			9				4		
17	42		836			1			9				4		

Legend: Nos (Numbers written in the color chart); X (Can’t read any number); Read Correctly (Number of children who read correctly); Misread (who missed the normal number); RGB no (Red Green Blind Number) → numbers that must be read by Red Green Blind Subjects; RGB read (number of children who read RGB numbers); and C. weakness (Color weakness)

As indicated in Table 3, for plate number 3 as an example, there were 811 students with normal color vision to read plate number 3 as 29 and there were 29 students who misread this plate. There were 8 red green deficient students who read plate number 3 as 70 instead of 29 and there were 2 individuals who were unable to read any number in this plate (color weakness). For chart numbers 16 and 17, there were 9 students who read plate 16 as 2 instead of 26, and plate 17 as 4 instead of 42. These students are strong deuteranopes. There were 4 students who read plate 16 as 6 instead of 26 and plate 17 as 2 instead of 42. These individuals are strong protanopes. There were 832 students who read correctly plate 16 as 26 and there were 5 students who misread this plate. There were 836 students who read plate 17 correctly as 42 and there was 1 student who misread it

The results of the multiple logistic regression analysis revealed that the variables’ sex, age and visual impairment were significantly associated with color blindness at 1% level of significant (*p* < 0.01) (Table 2).

## Discussion

Color blindness is an abnormal condition characterized by the inability to clearly distinguish different colors of the spectrum [4]**.** Color blind individuals may face difficulties at work as seen for technician working in color industries [7]**.** The prevalence of color blindness varies from race to race and differs in different geographical areas [5, 8].

In this study, the prevalence of color blindness among 850 school children was found to be 36 (4.24%). This was similar with study conducted in Ethiopia among male school children [3]. In a tudy conducted among care drivers in Addis Ababa, Ethiopia, the prevalence of color blindness was found to be 4.5% [13] which was inline with our study.

In our study, there was a highly significant association between sex and color blindness (AOR [95% CI] =3.19 [1.45; 6.98], *p*-value = 0.004). Males have 3.19 times higher chance of being color blind than females; which was similar with [7, 10]. In this study, there was a highly significant association between color blindness and visual impairment (AOR [95% CI] =4.15 [1.77; 9.75], p-value = 0.001). The chance of visually impaired children being colorblind is 4.15 times higher than the normal. This was similar with the idea reported by [14].

In this study, there was significant association between age and color blindness (OR = 0.8; 955 CI, 0.68–0.94; *P*<0.01). As age increases by one unit, the chance of being color blindness decreases by 20%. The reason may be the lower age range, more natural/inherited types/of color blindness in children than acquired type and recently in that area children may be born with inherited color blindness than the older one.

Studies showed that, the prevalence of color blindness varies from race to race and differs accross different geographical areas [5, 8]. Study conducted in India, the prevalence of color blindness was, 2.02%. Out of which, 3.16% were males and 0.4% female [7]**.** Another study in India, the prevalence was found to be 3.7% [8]**.** These findings were lower than our study.

In Iraq, 8.47% were colorblind [5]; in Philippines among male high-school students it was 5.17% and most common deficiency was the deutan type [10]. These were higher than our study. The reason for the difference in prevalence may be due to the difference in race, since mostly color blindness is inherited [5, 8].

## Conclusion

Color blindness was one of the public health problems in Gish Abay town district, Amhara Regional State, Ethiopia. The variables’ visual impairment and sex were significantly associated with color blindness. Screening of the children for vision at the time of school admission, periodic eye examination is recommended for early diagnoses and adjusting their occupation early in life. Ministry of Health, Ministry of Education and other stakeholders should give emphasis on the complications associated with color blindness.

## Abbreviations

AOR: Adjusted Odds Ratio
CI: Confidence Interval
CVD: Color Vision Deficiency
OR: Odds Ratio
